# Analysis of drug resistance in HIV protease

**DOI:** 10.1186/s12859-018-2331-y

**Published:** 2018-10-22

**Authors:** Shrikant D. Pawar, Christopher Freas, Irene T. Weber, Robert W. Harrison

**Affiliations:** 1 Department of Computer Science, 25 Park Place, Atlanta, GA 30303 USA; 2 Department of Biology, 100 Piedmont Ave., Atlanta, GA 30303 USA

**Keywords:** HIV protease, Drug resistance, Machine learning, RBM, Structure-based

## Abstract

**Background:**

Drug resistance in HIV is the major problem limiting effective antiviral therapy. Computational techniques for predicting drug resistance profiles from genomic data can accelerate the appropriate choice of therapy. These techniques can also be used to select protease mutants for experimental studies of resistance and thereby assist in the development of next-generation therapies.

**Results:**

The machine learning produced highly accurate and robust classification of HIV protease resistance. Genotype data were mapped to the enzyme structure and encoded using Delaunay triangulation. Generative machine learning models trained on one inhibitor could classify resistance from other inhibitors with varying levels of accuracy. Generally, the accuracy was best when the inhibitors were chemically similar.

**Conclusions:**

Restricted Boltzmann Machines are an effective machine learning tool for classification of genomic and structural data. They can also be used to compare resistance profiles of different protease inhibitors.

## Background

Human Immunodeficiency Virus (HIV) is a major pandemic disease [1]. More than 36 million people have been infected and about half of these people receive anti-retroviral therapy [2]. However, retroviruses like HIV mutate rapidly since the conversion from the RNA genome to DNA is error-prone [3]. They readily form quasi-species and distinct viral strains. Therefore, retroviruses can respond effectively to selective pressures such as drug treatment by mutating to evade the antiviral drug. The development of drug resistance in HIV is an ongoing threat to effective long-term therapy.

Machine learning can predict drug resistance from sequence data with high accuracy as shown by tests on genotype-resistance data for HIV protease and reverse transcriptase [4–11]. The critical improvement in the application of machine learning to drug resistance is the inclusion of structural data in the features. We found that using Delaunay triangulation to encode the protein structure [12] is highly effective. The encoding compresses a protein sequence and its corresponding structure into a feature set consisting of 210 components. The set contains the relative frequencies of each kind of amino acid pair from the structure. Yu’s use of compressed encoding in [4] suggested that even fewer features were necessary to encapsulate drug resistance. Therefore, we used Principle Components Analysis (PCA) to explore the remaining redundancy in the data. The availability of a large amount of sequence and resistance data for HIV protease (PR) has proved valuable for method development.

The validity of this approach was verified by experimental studies [13, 14]. Machine learning was used to rigorously select representative highly resistant PR sequences for biochemical and structural characterization. The computationally selected mutant demonstrated several orders of magnitude worse affinity for inhibitors compared to wild type enzyme. The selected mutant had only one mutation in the inhibitor binding site. Therefore, a high level of resistance was achieved almost exclusively by mutations distal from the active site.

Restricted Boltzmann Machines (RBMs) are a generative machine learning algorithm [15, 16]. RBMs only require positive, or in-class, training data, and often generalize more accurately than other approaches. Training the standard algorithm on large datasets is often computationally infeasible. We have developed a highly efficient version of this algorithm [17, 18]. Using a simplified representation of the hidden and visible spins and replacing a numerical estimate of the gradient with an analytic form results in an algorithm that is at least 14 times faster than the conventional algorithm without compromising the accuracy.

Generative machine learning has not been applied to drug resistance in HIV. Therefore, application of this approach to the analysis of drug resistance is of interest. This paper shows that RBMs are as accurate as other machine learning approaches for these data. Additionally, we studied how well RBMs trained on one drug were able to predict resistance for a different drug.

## Methods

### Datasets and data preparation

#### Datasets used for the study

The genotype-phenotype datasets were downloaded from the Stanford HIV drug resistance database [19]. Data were used for the HIV protease inhibitors: atazanavir (ATV), nelfinavir (NFV), ritonavir (RTV), indinavir (IDV), lopinavir (LPV), tipranvir (TPV), saquinavir (SQV), fos-amprenavir (FPV) and darunavir (DRV). All the datasets were pre-processed using the methods and the cutoff values described previously in [4]. The threshold for resistance recommended by the database curators was used in this work [19]. The results of the expansion of data for each of the HIV-1 PR inhibitors and proportion of resistant mutants are shown in Table 1.

**Table 1:** The results of the expansion for each of the HIV-1 PR inhibitors

Inhibitor	No. isolates	No. sequences	No. resistant	No. sensitive	Fraction resistant
SQV	1722	10258	4206	6052	41.0
DRV	607	5973	1889	4084	31.6
LPV	1444	10239	5095	5144	49.8
NFV	1771	10911	6170	4741	56.5
IDV	1730	10537	5122	5415	48.6
ATV	1141	8430	4237	4193	50.3
FPV	1681	10521	4405	6116	41.9
TPV	847	7363	2062	5301	28.0

#### Pre-processing/expansion of the datasets

Wild type HIV PR has a protein sequence of 99 amino acids. Sequences with insertions, deletions, or stop codons were removed. Genomic datasets often include multiple mutations at the same site. In these cases, the data were expanded to multiple sequences with single amino acids at each location to represent a single amino acid sequence for each mutant protein. For example, if one 99-amino acid mutant sequence has two different types of amino acids at one position and another site has three, this one sequence needs to be represented by six unique sequences each differing in only one amino acid substitution. The pre-processing method has been explained in detail in [4]. Each sequence was accompanied by its inhibitor resistance fold values. The relative resistant fold values for each of the inhibitors ranged from 0 to 800-fold resistance. Finally, the expanded datasets with sequences were allotted a unique identifier number to help recover the original sequences and their respective resistance fold change after analysis.

#### Encoding structure and sequence with Delaunay triangulation

A graph-based encoding system was utilized to represent the sequence and structural information of the protein [6]. The X-ray crystal structure for HIV-1 PR (3OXC) [20] was used as a template for creating the Delaunay triangulation. The structurally adjacent pairs of amino acids were represented as a vector of the 210 unique pairs of 20 standard amino acids. This graph-based encoding of sequence and structure has been proven to be a promising technique for fast and accurate predictions of resistance from sequence in HIV infections [5].

### Principal component analysis

Principal Component Analysis (PCA) using Singular Value Decomposition (SVD) was run on all the HIV-1 PR datasets using the Scikit-Learn machine learning library [21]. The datasets for each inhibitor were analyzed using the Pandas data analysis library [22]. The resistance fold values were not included in the PCA calculations since predicting these values is the goal of this work. The results of this analysis are detailed in the “Results” section.

### Training the RBM

The mutants with relative resistant fold less than 3.0 were classified as non-resistant (susceptible) and denoted as 0; while those with relative resistant fold of greater than 3.0 were classified as resistant and denoted as 1, as used in [4] and consistent with other analyses of the Stanford HIV resistance database [19]. RBMs work best with bit patterns. These bit patterns were generated by scaling and dividing the range of individual features into equal intervals. Each feature of the data was scaled to the range 0 to 1 based on the maximum and minimum values of that feature. The scaled data were divided into eight intervals encoded with three bits per feature. The testing and training sets were scaled independently.

The RBM was trained using gradient descent with the derivative as shown in Eq. 1. The analytic expression for the expected value of the derivative, shown in Eq. 2 and derived in [17], was used.

1 $$\begin{array}{*{20}l} &\frac{dU}{dW_{i,j}} = H_{j} V_{i} - <\frac{dU}{dW_{i,j}}>
\end{array} $$

2 $$\begin{array}{*{20}l}  <&\frac{dU}{dW_{i,j}}> = H_{j}\frac{e^{\beta U} - e^{-\beta U}}{e^{\beta U} + e^{-\beta U}} \end{array} $$

In these equations, H and V are hidden and visible (or input) layers respectively, *β* is the inverse temperature, U is the potential energy, and W are the weights used to define the potential.

During training, the layer that gave the best fit for each new data point was updated with a descent step and the other layers were “anti-trained” with a small ascent step. “Anti-training” improves the convergence and training efficiency of the RBM. Anti-training is only feasible when using an analytic expression for the training gradient. An RBM with 150 units in the hidden layer was trained for each category with a constant step-size of 0.1. A step-size of 0.01 was used for anti-training. An RBM was trained for both resistant and non-resistant classes. Class membership was assigned by the fractional reconstruction error, shown in Eq. 3 as defined in [17].

3 $$ \begin{aligned} R = \frac{H_{i} {\sum}_{j} W_{i,j} V_{j}}{\left|H_{i}\right| {\sum}_{j} \left|W_{i,j}\right| \left|C_{j}\right|} \quad \text{where C is the perfect reconstruction.} \end{aligned}  $$

Five-fold cross validation was used to ensure that the results reflect the error in the models. The models for each fold were trained to convergence with ten iterations and the values for accuracy, positive predictive value, recall, and F from the last iteration were reported.

## Results

### Classification with an RBM

The classification results are detailed in Table 2 and show a high degree of accuracy. The nearly uniform values of close to 1.0 for accuracy, PPV, recall, and F-score, show that the models reliably predict both resistant and non-resistant classes. These results compare favorably with our earlier results using non-generative machine learning algorithms [4–6, 8].

**Table 2:** The accuracy of the machine learning model is shown here

Inhibitor	Accuracy	PPV	Recall	F
Idv	0.979	0.974	0.985	0.979
Lpv	0.984	0.977	0.992	0.984
Sqv	0.969	0.963	0.986	0.974
Tpv	0.987	0.984	0.998	0.991
Drv	0.988	0.985	0.998	0.992
Atv	0.983	0.976	0.989	0.983
Nfv	0.978	0.974	0.975	0.975
Fpv	0.988	0.984	0.998	0.991

The estimated standard deviation amongst the five folds is <0.013 for all values

### Cross-classification with an RBM

RBMs differ from non-generative machine learning methods in an interesting way. It is trivial to train an RBM against one dataset and use it to predict the behavior of another. Table 3 shows the results of a cross-training analysis of resistance data. Each row was trained on one inhibitor and the columns show the accuracy with which that model predicts the resistance for the other inhibitors. The inhibitors generally, but not completely, cross-classify with high accuracy. TPV and DRV seem to have more differences from the other inhibitors.

**Table 3:** Cross training reveals similarity between the inhibitors

Compound	Atv	Drv	Fpv	Idv	Lpv	Nfv	Sqv	Tpv
Atv	0.990	0.868	0.880	0.955	0.946	0.914	0.893	0.819
Drv	0.767	0.996	0.818	0.786	0.785	0.718	0.792	0.925
Fpv	0.929	0.873	0.981	0.889	0.886	0.822	0.822	0.828
Idv	0.945	0.863	0.880	0.989	0.960	0.905	0.878	0.809
Lpv	0.939	0.892	0.877	0.963	0.988	0.891	0.865	0.837
Nfv	0.923	0.853	0.824	0.918	0.901	0.987	0.837	0.758
Sqv	0.898	0.837	0.825	0.890	0.871	0.840	0.983	0.807
Tpv	0.723	0.929	0.765	0.729	0.728	0.655	0.732	0.993

These numbers show the accuracy when a model trained on the compound at the start of the row is used to classify the data from the other inhibitors

The ability of an RBM trained on resistance to one inhibitor to predict the behavior of resistance to another inhibitor shows that the drug resistance of HIV protease does not fully depend on the type of drug. The existence of cross-resistance is well known and our lab has used similar approaches to identify interesting multi-drug resistant mutants for structural study [6, 13, 14].

### Principal component analysis

**Fig. 1:**
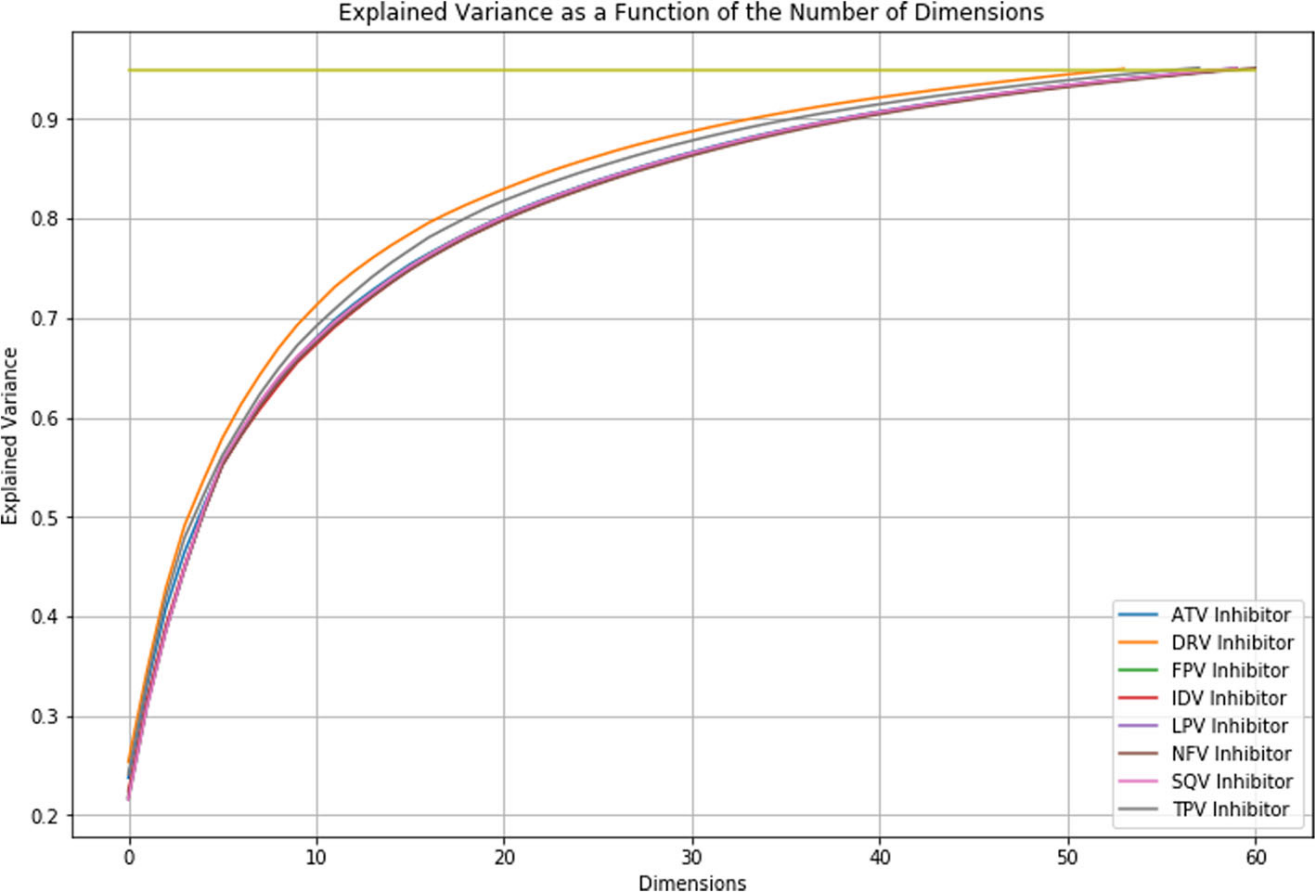
Principal Component Analysis on the HIV-1 PR Datasets. The similarity in the curves indicates that the datasets have a similar underlying structure

Figure 1 shows the explained variance for each of the datasets as a function of the number of reduced dimensions. As shown in the figure, there is overlap between some of the datasets (some of the plots depict the same data). This suggests that redundancy exists between datasets and not just within a single dataset. The horizontal line in the figure depicts where at least 95% of the explained variance of the datasets is captured. In most cases, the first principal component explained at least 90% of the observed variance. This was true for the ATV, LPV, NFV, and SQV inhibitors. The remaining four inhibitors had an explained variance ratio between 51% and 87% for the first principal component. For all inhibitors except for DRV, 95% of the explained variance for each dataset was captured within 60 dimensions, suggesting that the data could be further compressed while still minimizing the reconstruction error. For DRV, the explained variance could be reduced to 50 dimensions. These results indicate that a more compact encoding for the resistance data exists, consistent with Yu’s results on effectiveness of compressed encoding for machine learning [4].

## Discussion

### Classification of resistant mutations of HIV PR

The combination of structure-based encoding and RBMs is an effective technique for the prediction of drug resistance in HIV PR. The five-fold cross validated results in Table 2 clearly demonstrate their success and accuracy. The high values for PPV indicate that the models could be clinically valuable. The use of an RBM is especially interesting because there are essentially no adjustable parameters in the process. Efficient training algorithms allow the RBM to handle large datasets in reasonable times. While these datasets are not quite big data, they are too big for other machine learning programs [7, 23].

#### Comparison with other methods

We pioneered the approach of using a unified representation of sequence with 3D structural data expressed as a 210-long feature vector for machine learning [4]. This approach gave improved accuracy for predicting drug resistance for HIV protease and reverse transcriptase compared to using sequence data alone.

Another group reported mean R2 values of >0.95 for regression with ANN using a subset of HIVsequences restricted to subtype B with the data filtered to remove rare variants [10]. Their classification accuracy was less impressive. Structural data can also be represented by molecular mechanics calculations on protein-drug complexes. Molecular interaction components calculated between a drug and 36 single mutants of HIV protease were used for SVM classification of resistance and showed improved accuracy over using sequence alone [9]. These results were comparable to our earlier results, but for a much smaller number of sequences. Feature vectors derived from a four-body statistical potential and n-grams were applied in [11]. This approach also used explicit atomic models for the protease and therefore only a few hundred mutants were included for classification and regression. Their reported accuracy was worse than ours.

Our approach preserves structural information using Delauney Triangulation derived from a single protein structure, and is applicable to any mutant, while eliminating the expensive step of calculating molecular properties for models of every mutant structure.

#### Redundancy in the data.

One of the original motivations for exploring graph-based encoding of protein structures was to remove unnecessary data while retaining the critical features for machine-learning based analysis of structure and function [12]. Earlier work [4], which used compressed encoding, hinted that the redundancy was not completely removed from the data. Our use of PCA on the data demonstrated that further compression is possible because the majority of the variance in the data could be captured with 50–60 dimensions instead of the 210 used in the original representation. This strongly suggests that we may be able to extract patterns of mutations associated with drug resistance from the structural data itself.

### Inhibitor specific patterns of drug resistance

**Fig. 2:**
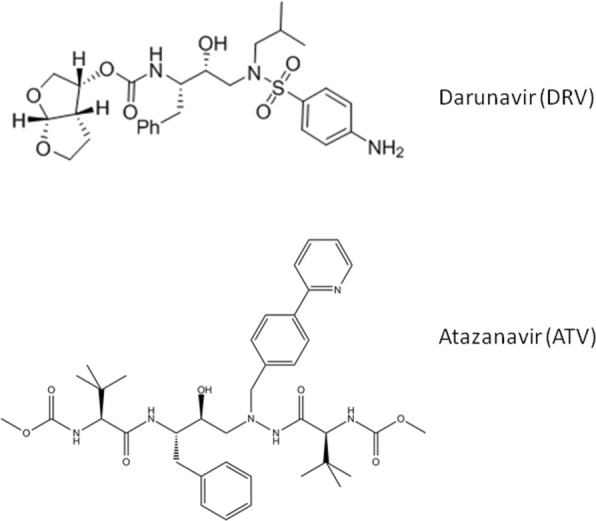
The chemical structures for a sulfonamide-containing (DRV) and non-sulfonamide-containing (ATV) inhibitor are shown here. These demonstrate the variety of chemistry used in inhibitors

Another important difference between generative machine learning and more conventional algorithms is that it is logically consistent to apply generative machine learning across categories. Since the RBM is essentially measuring how well it can reconstruct a given data point, it makes sense to ask whether an RBM trained on one inhibitor such as ATV could reconstruct data for a different inhibitor such as DRV. Examples of two inhibitors, Darunavir and Atazanavir are shown in Fig. 2 which demonstrates the diversity of drug chemistry used to inhibit HIV PR.

The inhibitors segregate into two main classes in the cross-training analysis. Cross-training results in high accuracy for most inhibitors, with the exception of DRV and TPV, which both incorporate sulphonamides. DRV and TPV, predict each other with reasonable accuracy (92.5%), however they show worse prediction for other inhibitors. While this could be due to chemical similarity, it could also be due to these being second generation or salvage inhibitors where the full spectrum of resistance mutations has not had time to evolve.

Accurate cross-prediction is not completely surprising. The inhibitors bind to an active site that is under selective pressure to still recognize its biological substrate. Many of the most highly resistant strains demonstrate multi-drug resistance [6, 13, 14, 24]. Therefore, we expect some level cross-prediction and this work quantifies it.

## Conclusion

Generative machine learning algorithms such as the RBM are well-suited to the prediction of drug resistance in HIV PR, and likely will work on other systems as well. The graph-based structure/sequence encoding used in this and related work removes much of the redundancy in the data, but does not remove it all. This result suggests that even more efficient encoding schemes are possible. The RBM was used to analyze similarities in resistance profiles for different clinical inhibitors. The analysis suggests that there are at least two main classes of inhibitors for HIV PR.

## Abbreviations

ATV: Atazanavir
DRV: Darunavir
FPV: Fos-Amprenavir
HIV: Human immunodeficiency virus
HIV PR: HIV protease
IDV: Indinavir
LPV: Lopinavir
NFV: Nelfinavir
PCA: Principle components analysis
PPV: Positive Predictive Value
RBM: Restricted boltzmann machine
SQV: Saquinavir
TPV: Tipranavir
